# An exploration of the knowledge, attitudes and beliefs of Xhosa men concerning traditional circumcision

**DOI:** 10.4102/phcfm.v9i1.1454

**Published:** 2017-10-13

**Authors:** Salome Froneman, Paul A. Kapp

**Affiliations:** 1 Division of Family Medicine & Primary Care, Stellenbosch University, South Africa

## Abstract

**Background:**

The practice of traditional circumcision is associated with considerable morbidity and mortality, yet there is a paucity of literature that provides an understanding of the cultural values that influence men to choose traditional rather than medical circumcision.

The aim of this study was to better understand the culture surrounding traditional circumcision, with a view to addressing morbidity and mortality rates associated with the Xhosa male initiation rituals.

We explored Xhosa men’s perceptions regarding the need for the risks and the social pressure to undergo traditional circumcision, the impact of non-initiation or failed initiation and the perceived barriers to obtaining medical help for the complications of traditional circumcisions.

**Methods:**

Individual in-depth interviews were conducted with 10 purposively sampled teenagers and adult men. The interviews were recorded, translated, transcribed and analysed using the framework method.

**Results:**

Traditional circumcision was seen as essential to Xhosa culture. Participants rationalised many reasons for participating, including personal growth and development, family and peer pressure, independence and knowledge gained, a connection with ancestors and initiation into manhood. Despite publicity of the dangers of traditional circumcision and the hardships they have to endure, most young men still saw this process as necessary and worthwhile.

**Conclusion:**

Traditional initiation and circumcision are here to stay. The majority of boys still trust the elders and supernatural processes to guide them. However, some participants welcomed government initiatives to reduce human error causing unnecessary death and suffering. Current systems to prevent morbidity and mortality are insufficient and should be prioritised.

## Introduction

Traditional Xhosa male circumcision rites need to be understood within a highly complex socio-sexual context involving violence, family breakdown, resource scarcity and inter-generational conflict.^1^ Traditional circumcision may be seen as a sacred religious practice,^2^ has been rationalised as a mechanism for the maintenance of social order^3^ and is believed to play a role in the prevention of HIV.^4^ The manhood status achieved after the ritual accords men power and authority in the community over women and uncircumcised men.^5^ As such, it is important for Xhosa men to be circumcised. Significant stigma is attached both to failed initiates and uninitiated people. Boys have to be successfully initiated to marry, inherit property or participate in cultural activities such as offering sacrifices and community discussions.^6^ If they are not circumcised, they are given leftover food at celebrations, are not allowed to socialise in taverns with other men, are not allowed to use the family name to introduce themselves and are sometimes forcefully taken away from their girlfriends.^6^ Uninitiated men have less autonomy and must often obey others.^7^ They are accused first in the event of theft because ‘only boys steal’ and are often subjected to public humiliation and name calling. They are seen to be cowards who do not respect their culture and would incur the wrath of the ancestors for not complying with cultural expectations.^6^ Successful initiates have two ways to prove and defend their manhood: firstly, through the new language and riddles taught by elders during initiation and, secondly, by the circumcision scar (not the ‘cat claw’ scars left by sutures inserted during medical circumcision).^6^

The complications of traditional circumcision were quantified in a study in the Eastern Cape during 2010 and included sepsis (56.2%), genital mutilation (26.7%), dehydration (11.4%) and amputation of genitalia (5.7%).^8^ Depression commonly follows complications of traditional circumcision.^6^ The South African National AIDS Council (SANAC) reported 32 deaths (25 in the Eastern Cape, 5 in Mpumalanga, 1 in the Western Cape and 1 in Limpopo) occurring nationally during the 2014 winter initiation season. Despite increased government initiatives to close down illegal initiation schools, the Eastern Cape Department of Health reported 32 initiation deaths and more than 144 hospital admissions for the 2015 winter initiation season.

Factors contributing towards morbidity and mortality during traditional circumcision include using the same knife for the whole group, voluntary dehydration and ‘ukumetsba’. ‘Ukumetsba’ is where the initiate has sexual intercourse with a girl whom he does not intend to marry and whom he will ‘hate’ afterwards.^7^ Fatal clashes between traditional nurses and community members have also been reported.^4^ The ritual is a test of manhood, and pain and bravery are essential components of this test. Seeking medical help is considered taboo and results in failed initiation.^3^ The community perception is that death during initiation is a way for the ancestors to point out who would never have been real men anyway.^9^ During the Health Department’s campaign against illegal circumcision schools and illegal traditional surgeons in 2006, one chief criticised the Department for interfering. His opinion was that the ongoing deaths showed that the ancestors did not approve of what health officials were doing.^4^

In the former Transkei, 67.0% of initiates were unaware of the risks of traditional surgery in 2005.^3^ In 2010 it was found that mothers of initiates preferred hospitalisation for circumcision, although there was agreement that traditional initiation is a prerequisite for becoming a man.^10^ This preference may indicate a possible transition phase where people are more aware of the complications of traditional circumcision and safer circumcision is becoming more desirable. Also, Nwanze and Mash demonstrated a community commitment to safer circumcision in Umlamli, Eastern Cape, in 2012.^11^

This study aimed to explore the cultural values relating to circumcision in a changing society and how Xhosa individuals weighed up the risks and benefits within their broader community context. Although previous studies focused mainly on the rural Eastern Cape, this study was conducted in the Western Cape, where there is a dearth of data on morbidity or mortality as a result of circumcision. We focused on the perceptions of contemporary Xhosa men, exposed to media reports of complications of traditional circumcision, with free access to medical circumcision and influenced by other possible indicators of success and status, such as money and education, as a result of urbanisation. An increased awareness of these factors might open a platform for the discussion of safer traditional circumcision practices in future.

## Research methods and design

### Study design

This was a phenomenological qualitative study using individual semi-structured interviews.

### Setting

The Knysna and Bitou sub-districts of the Western Cape border on the Eastern Cape, forming a transitional zone between deep rural and semi-rural communities. The community of approximately 114 000 is a mixed race group (41% black African)^12,13^ with population growth between 5% and 9% annually, mainly because of influx from the Eastern Cape. The African population is estimated to have grown by 200% in the past 10 years.^2,13^ Most are Xhosa speaking, and many still follow traditional customs.^13^ During the circumcision season, many initiates are seen in the local clinics and the district hospital as a result of complications related to circumcisions. These Xhosa initiates often find themselves mutilated as a result of sepsis and some require penile amputation.

The research assistant was a young male Xhosa nurse who resides in Knysna and is a respected health professional in the Knysna Hospital and originates from the Eastern Cape. He is relatively unknown amongst the Xhosa community of Bitou and Knysna. He understands Xhosa culture and has undergone initiation himself. He was trained to do qualitative interviews. He was chosen to overcome the cultural, gender and language barriers posed by the researcher’s demographics: a white, female, Afrikaans speaking doctor concerned with the high incidence of complications related to traditional circumcision, but who lacked knowledge of Xhosa traditions and circumcisions.

### Selection of participants

Purposive sampling was used to select a variety of suitable participants. The participants were all Xhosa men residing in the Eden district, at least 18 years of age or older, and either already initiated or eligible for initiation. A mix of men who actively engaged with Xhosa traditional healers and ceremonies or who embraced allopathic medicines and ideas were selected.

Men with failed circumcision were excluded by the ethics committee. Data saturation was decided to have been achieved if no new themes or codes were identified. Sampling continued until data saturation was reached. This was found to be at the eighth interview. A further two interviews were conducted to confirm data saturation with no new themes or codes being identified.

The research assistant identified the possible participants with the help of a male nurse who is active in the Xhosa community of the Knysna and the Bitou sub-districts based on the above criteria as determined by the researcher.

### Data collection

The research assistant approached the participants for consent and conducted the interviews. Selected participants were interviewed at their houses or their nearest primary care clinic in a private consultation room. Individual semi-structured interviews were conducted using an interview guide (see Appendix 1). The participants’ views on Xhosa tradition, circumcision and its relevance today, dangers of traditional circumcision and the role that modern medicine has to play were explored. The interview guide served as a template to ensure all relevant topics were explored, but did not exclude the possibility of exploring unanticipated issues that emerged in the interviews. Interviews ranged from 45 to 60 minutes each and were undertaken in English or Xhosa depending on the preference of the participant. Interviews were audio recorded. The interviews were transcribed and translated by a translator fluent in English and Xhosa, and who understood Xhosa traditions and customs. The transcription was done verbatim while ensuring that the original meaning was not lost during the translation. The background details of the participants collected by the interviewer were added to the transcripts.

### Data analysis

The transcribed and translated data were analysed using the framework method. The researcher immersed herself in the data. A thematic index was developed by coding the data and organising the codes into categories. The transcripts were indexed by systematically applying the codes to all the data. Charting was done by bringing together all data with the same codes. These were interpreted to identify the range and depth of themes and any relationships between them. Memos (field notes) and a reflexivity journal were kept and used to assist in the analysis.

### Ethical considerations

The research was approved by the Health Research Ethics Committee of Stellenbosch University (Reference S14/07/148) and the provincial government of the Western Cape (Reference number: WC_2015RP40_255). The study was conducted according to the ethical guidelines and principles of the International Declaration of Helsinki, South African Guidelines for Good Clinical Practice and the Medical Research Council Ethical Guidelines for Research.

## Results

The participants interviewed are described in Table 1.

**TABLE 1:** Demographics and characteristics of the participants.

Participant	Age (years)	Occupation	Marital status	Circumcised or not?	Making use of allopathic medicine	Actively practising Xhosa traditions
a	18	Learner	Single	No	No	Yes
b	27	Unemployed	Married	Yes	No	No
c	23	Painter	Married	Yes	Yes	Yes
d	18	Learner	Single	Yes	Yes	Yes
e	19	Learner	Single	No	No	Yes
f	20	Gardener	Single	Yes	No	Yes
g	34	Teacher	Married	Yes	Yes	No
h	22	Student	Single	Yes	Yes	Yes
i	26	Unemployed	Single	Yes	No	Yes
j	31	Labourer	Married	Yes	No	Yes

*Source*: Authors’ own work

The following major themes were identified:

- Respect is earned.
- A boy forever.
- Becoming a man.
- Bush medicine is best.
- Faith in the elders.

### Respect is earned

Traditional circumcision was seen as essential to Xhosa culture. Participants gave many reasons for partaking in traditional circumcision practices, including family and peer pressure, knowledge gained, as well as a connection with ancestors and initiation into manhood. Respect earned in the community was by far the most frequently mentioned reason:

‘You will no longer be a boy; instead everyone will listen to what you have to say because you are no longer a boy.’ (Participant e, 19, Learner)

#### Personal development and belonging

The curiosity, enigma and camaraderie surrounding the practice were difficult to verbalise. A sense of belonging was created. Some boys were enthusiastic and approached their parents to request them to send them to initiation school:

‘The thing that encourages me is the traditional way when there is a ritual in the house, and you see men there having fun … something like that … and I said to myself: “Hey, one day I will like to be among those guys, having fun there.” ’ (Participant c, 23, Painter)

Participants felt incomplete without undergoing this process:

‘When you come back, you will be right. That is why I need to go, to finish this thing. Otherwise, I won’t be a man.’ (Participant e, 19, Learner)

Apart from being taught how to behave respectfully, boys also gained respect by successfully undergoing the traditional circumcision rituals. After undergoing circumcision, they were included in the cultural activities and consulted in family decisions. Traditional circumcision rituals were said to connect them with their ancestors.

#### Humiliation and shame

Boys who refused to go or who failed the traditional circumcision ritual, including those obtaining medical help, were described as bringing ‘humiliation and shame’ to the family that resulted in the parents suffering emotional turmoil because they realised that their child would have to endure many hardships because of this. They felt that they had failed in bringing their child up with good values and were concerned about who would take care of the family when they became old and died.

Parents and the community went to great extremes to try and convince children to go for traditional circumcision. If a boy did not listen to his father, other family members and elders would try to convince him:

‘If I have sons, they must go, because my forefathers and my father’s father and my father and I also went through the process. If they don’t go, it will be a humiliation to me, and they will throw tradition back to my face. They will have to go. There is no other way.’ (Participant h, 22, Student)

Some parents refused to sign the consent forms for medical circumcisions for boys under the age of 18 years, as they felt these children were moving away from their traditional responsibility. Some even forced traditional circumcisions on their sons.

Some boys were kicked out of their parent’s houses and left to ‘see for themselves’ for choosing a medical circumcision. However, parents were willing to go to great lengths to force their children to undergo a traditional circumcision included the use of coercion and even physical force:

‘The thing is they must go. If they refuse, I will not let them refuse. I will beat them.’ (Participant h, 22, Student)

#### Ancestral connectivity

Traditional circumcision was seen as an obligation to the ancestors that needed to be fulfilled by all Xhosa boys. Parents whose children refused to undergo the traditional rituals had to apologise to the ancestors:

‘It is necessary for our black people because it connects us with our ancestors and stuff, you know. …’ (Participant f, 20, Gardener)

### A boy forever

An initiate who had had an incomplete transition into manhood was called by his name and not ‘*bhuti*’ (brother). Often names like ‘coward’ or ‘*inkwenkwe*’ (boy) were used, even by children in the community. Men who had not completed the traditional initiation were not allowed to socialise or stay with the successful initiates when they came back and were excluded from traditional ceremonies. They were excluded from the bonds formed during the initiation process and were often ridiculed, as the other initiates and the community might feel that the uninitiated person was not transparent in his intentions and integrity:

‘He is going to suffer a lot. He will never be a man to the Xhosa people.’ (Participant g, 34, Teacher)

The interviewees believed that a dark cloud of bad luck or ‘ibhadi’ would follow those who did not undergo traditional circumcision. If an initiate does not get taught the ‘isidoda’ or ‘the language of manhood’ at initiation, he cannot be a man.

Some believed that if your younger brother had a traditional initiation before you, you have to respect him and treat him like he is the older brother. If you went to the clinic and turned your back on the tradition, the community would treat you the same and turn their back on you. There was also no way to go back on your decision and have a traditional initiation after a medical circumcision. You had to live with the consequences of your decision for the rest of your life:

‘Yho the problems are big. The problem is maybe there is a ceremony at your house, in your own home. Maybe your father is making some traditional thing, and then the successful initiates go to the ceremony and chase you out of your own home and say: “Go play outside because you are still a boy”. That will hurt a lot. You could go on and say that this is my father’s home, but even your father will convince you that you are just a boy and that you must leave.’ (Participant f, 20, Gardener)

Education and money can be substitutes for the respect earned by traditional circumcision, but will not replace the sense of belonging that traditional circumcision creates. Neither will he be allowed to attend the traditional ceremonies:

‘When you have a medical circumcision as a Xhosa, and you are highly educated, that can really protect you, because in Xhosa we really respect the one who comes here with his BMW. They won’t even remember if he has gone to the clinic or the bush. But he can’t go to the ceremonies because the traditional things are so secretive, you see.’ (Participant g, 34, Teacher)

### Becoming a man

Together with the newly earned respect came more responsibility. The initiates had to be more punctual, had to take more initiative and were more aware of their duties in society. Becoming more mature is not something that happened instantly, but there is an appreciation that you now have to be an example to other boys and start picturing yourself as an elder to those who are still boys.

One participant said that he thought that he would have more freedom after initiation and fewer limits to what he could or could not do. However, after initiation, he felt less free because of all the new responsibilities.

On the other hand, family members no longer ask him for favours or give him chores; he now has the authority to assign tasks:

‘I would maybe sleep a lot and watch TV, but now that I’m a man I must wake up on time and do all those things that need to be done at home.’ (Participant a, 18, Learner)

A whole new world opens up concerning inclusion into the secrets and plans of the family. Their social status also increased significantly and they were allowed more quality time with adults:

‘My father is a very quiet man, and I never heard him speak to me like this, he would just greet me and ask how I was and then send me somewhere, but now I can sit with my father and talk and share. My father is a very old man, and he knows a lot about relationships, and I want him to explain those things to me.’ (Participant d, 18, Learner)

One participant was given a house after initiation:

‘They are looking up to me now, and they [*are*] making me like I’m one of the presidents.’ (Participant a, 18, Learner)

### Bush medicine is best

#### Knowledge transferral

Boys are taught how to behave like men, how to manage themselves and how to understand other people and relate to community members of different ages, including children and parents under different circumstances. Furthermore, they are taught how to look after their houses and livestock. They are informed about social norms including what time to come home in the evening and how to accomplish their duties. Independence, authority and responsibility for daily activities are reinforced. The initiation process marks the transition from using their free time for playing to using it to improve themselves:

‘I learnt how to do the things of men, you know, like be a man in my house … also what do with the cows.’ (Participant a, 18, Learner)

#### Medical help

Although boys were encouraged to visit the clinic to exclude any illness before the circumcision, receiving medical assistance during the ritual was strictly forbidden and seen as cheating. Complications of traditional circumcisions were seen to be because of pre-existing medical conditions for which initiates were not being treated during the initiation period.

If a boy phones an ambulance to fetch him while in the bush or obtains any medical help during the initiation process, he would not be allowed to complete the initiation ritual and would be seen as having failed initiation. It is even more problematic if the boy sees a female healthcare worker at the clinic or hospital. Sometimes these kids are treated similarly to those who refused to undergo traditional circumcision and become outcasts in the community:

‘That is a big, big NO! Pain pills and injections and all those things are not allowed here. Bandages are not allowed here. Even Zambuk and this Real Makoya stuff is like a hospital in your pocket. You see – it is a traditional circumcision – there is a traditional healer for those things.’ (Participant b, 27, Unemployed)

#### Traditional help

Traditional healers were available to assist with health concerns during traditional circumcisions. Traditional medicines were used to treat the ailments and infections reported to them:

‘There are people who can help, but you can’t nag, and you can’t phone the ambulance. They know what to do, they were in the situation before.’ (Participant b, 27, Unemployed)

#### Human immunodeficiency virus

Disclosure of HIV status and infection control was found to be problematic. Some initiates were under the impression that all boys undergoing traditional circumcision were HIV negative and were therefore not infectious:

‘You are supposed to go through the clinic to see that you don’t have HIV. Just because at the bush they use their hand to help you with no gloves. So you must check yourself, and when you are HIV-infected you will not be helped, and you will remain a boy for the rest of rest of your life’. (Participant b, 27, Unemployed)

Not only are gloves not used, but one initiate explained that during his initiation one knife was used for multiple circumcisions:

‘They were not careful – you can’t use one knife for five people because you don’t know that the other person got, who you began with, and they forget to wipe the blood off. …’ (Participant f, 20, Gardener)

#### Trust

Participants had faith in the correctness of the procedures performed by elders. One interviewee said that the reason why one of his fellow initiates died was that he did not listen to the elders and did not trust them. Another initiate’s father was a taxi owner and very wealthy and powerful. The father influenced some of the elders to give him special treatment to *‘*treat him like an egg’ – he had a cell phone there, which was not allowed. During the first seven days when they were not authorised to drink any water, he was secretly drinking ‘Sparletta’. This boy eventually got sick and was taken to hospital:

‘So you must not judge whether they wrong or right, you must just listen to what they say in order to succeed. They know everything from there … you must just follow their experience.’ (Participant c, 23, Painter)

### Faith in the elders

Most participants felt safe in the presence of their friends and confident in the elders. They felt that no changes were necessary to improve safety:

‘As long as you obey the elders, nothing could go wrong.’ (Participant d, 18, Learner)

In the cases where complications had developed, it was thought to be because of non-compliance or disapproval by the ancestors. One participant mentioned that some elders were unclear about the rules and made them suffer unnecessarily. This punishment made it difficult to succeed, especially as the elders were supposed to be more supportive and guiding during this difficult time:

‘If you don’t listen, they strike you down. Some will strike you down till you can’t go on and even when they don’t tell you what you must do they can strike you.’ (Participant a, 18, Learner)

#### Media cannot be trusted

Some participants were aware of complications of traditional circumcisions and media reports of deaths, but thought that it should not be published because of the adverse effect it may have on boys that still need to undergo the initiation in future.

Other participants felt that media warnings of deaths following traditional initiation were false and were fabricated to scare boys and prevent them from undergoing traditional circumcision. They believed that boys become even healthier and stronger during initiation and that these rumours were being spread by people undermining the Xhosa culture and who want the culture to come to an end:

‘You can’t get scared of something that you have never seen.’ (Participant f, 20, Gardener)

Media reports were seen as negative and fear-instilling tactics that should be ignored.

One participant said that he believed that media reports could be true, but that he would not speak about it further. This silence raises the concern that Xhosa people may be forced to deny some facts and develop a blind loyalty towards initiation practices:

‘There may be some risk, but if you want to be a man, you will just take the risk because you want this.’ (Participant e, 19, Learner)

#### A call for legal guidance

More than one participant wished to eliminate dangerous and harmful practices, by standardising the process and having the procedure done by experienced leaders who want them to succeed, consider individual needs and do not make them suffer unnecessarily:

‘Some punish you. They don’t even give you the rules; they just make you suffer.’ (Participant a, 18, Learner)

Participants were willing to undergo pain and risk, but also wished for the safest possible circumstances that eliminated human error as far as possible. One interviewee wanted the government to use the law and ensure that initiation schools were registered and to remove illegal schools:

‘I can say in my camp there were people who don’t have papers. They must change that. They must strictly change that. Because we are in a new generation, things must go according to the times.’ (Participant g, 34, Teacher)

## Discussion

This study clarified key social and cultural reasons for young Xhosa men choosing to undergo traditional circumcision. They expect to reap rewards of belonging, camaraderie and to achieve a new status as a ‘man’ in the family and community in contrast to just being a ‘boy’. Education and money may be substitutes for the respect earned by traditional circumcision, but may not achieve the sense of belonging that traditional circumcision creates.

Complications of traditional circumcisions were denied by some initiates who struggled to link septic wounds that developed later to the traditional circumcision process and wound care. One sensed the boys’ unquestionable faith in inherited procedures and willingness to protect their cultural habits. Studies from other regions had similar findings where initiation deaths were attributed to supernatural causes rather than human processes.^9,14,15^ However, in our study participants were starting to raise concerns about the actions of the elders and the registration of initiation schools. There appears to be a shift towards recognising a responsibility to eliminate unnecessary risk and ensure that initiation schools are registered and capable of performing safe procedures. Although government intervention was still unwelcome in 2006,^4^ some initiates from our study thought that the government must intervene to reduce circumcision deaths. This finding was confirmed by Nwanze who reported on a community in the Eastern Cape that was willing to collaborate with the health system to mitigate the risk.^11^ The Application of Health Standards in *Traditional Circumcision Act* (no. 6 of 2001) was welcomed in this community.

The literature informs us that death rates have not been substantially reduced from 1996 to 2015.^7^ Participants in this study confirmed that the same knife is still sometimes used for multiple individuals. This seemingly persistent practice in at least some of the initiation schools raises concerns regarding the efficacy and scale of government interventions to train traditional surgeons and close down illegal initiation schools. This practice has been outlawed in the Eastern Cape by the Application of Health Standards in *Traditional Circumcision Act* (no. 6 of 2001); however, this *Act* does not apply in the Western Cape.

In an attempt to reduce the deaths and complications of botched circumcisions the researcher recommends that the Western Cape Government should introduce legislation similar to the *Eastern Cape Act* no. 6 of 2001. There should be better policing of illegal initiation schools, and the community should be encouraged to report illegal schools. Unregistered traditional surgeons should be prosecuted under the *Traditional Health Practitioners Act* (no. 22 of 2007). High schools should distribute lists of legal initiation schools that have trained traditional doctors and that have a good track record. Increased opportunities for training of traditional surgeons should be made available. The Department of Health needs to reach out to schools and communities to raise awareness of preventable complications of traditional circumcision. Better relationships between healthcare workers and initiations schools may lead to quicker consultation and referral. The ideal would be shared management, where traditional and allopathic medicine are not mutually exclusive. An intervention similar to that done in Umlanili^11^ needs to be put into place in areas in the Western Cape in areas where traditional circumcision is practised. Training traditional surgeons in aseptic technique and providing them with sterile equipment should reduce the sepsis rates and risk of HIV transmission.

This study must be seen through the lens of the researcher and her demographics, a white, female doctor who could not interview participants directly, as it is inappropriate for women to discuss circumcision with men in the Xhosa culture. Some interviews were cut short by the interviewer without exploring relevant material in more detail and depth. Men with failed traditional circumcision were excluded from this study. This exclusion has led to a limited understanding of the hardships they endure or the resolute lives they may lead despite a failed traditional circumcision. Exploring their experiences may be the focus for further studies.

## Conclusion

From this study, it is clear that traditional initiation and circumcision is here to stay. The Xhosa people still trust the elders and supernatural interventions to guide them through the process. However, some participants were welcoming government initiatives to regulate the practices so as to reduce unnecessary death and suffering during initiation and circumcision. Current systems in the Western Cape to prevent morbidity and mortality are lacking rigour and should be prioritised, with attempts to improve collaboration between traditional and allopathic medicine. Although some efforts may have been useful in raising awareness of complications, they have not significantly reduced complications or deaths. Illegal initiation schools and unregistered traditional surgeons continue to operate. The time may be ripe for the local governments to review the concept of traditional initiation and transform it into a safer, contemporary practice without sabotaging the essence of this important practice.

## Appendix 1

### Discussion schedule

Use consent form to establish voluntary participation and understanding of aims of the study. Make sure that the participant is relaxed and ready to tell his story.

- Opening question

Medical doctors who are doing medical circumcision would like to know more about traditional circumcision. Can you tell me what your thoughts are concerning traditional circumcision for Xhosa men?

- Personal experience

Now that you know a bit more about the study, tell me about yourself? Allow the interviewee time to relax and elaborate on his views and experiences regarding traditional circumcision. Prompt interviewee to tell more.

- The importance of traditional circumcision

Why is it important for you to have a traditional circumcision? What problems would transpire if you chose not to have the traditional initiation?

- Morbidity and mortality

Explore any concerns about traditional circumcision.

Do you know of anyone who developed problems during or because of traditional initiation? Why do you think this happened? How would one go about obtaining medical help for problematic traditional circumcisions?

- Western culture

Explore how Westernisation is influencing traditional circumcision.

Do you think people are more careful and reluctant to undergo traditional circumcision than in the old days? In what way does prior medical circumcision influence traditional initiation?

- Influence

Explore who influences the decision to have a traditional circumcision.

What part does the opinion of your family, friends, co-workers, elders, media and science play in your decision?

- Before and after

Explore the experience after circumcision.

Tell me about how your life changed after the traditional initiation? Was this what you expected to happen? Would you want your children to be traditionally circumcised?
